# Viscoelastic lithography for fabricating self-organizing soft micro-honeycomb structures with ultra-high aspect ratios

**DOI:** 10.1038/ncomms11269

**Published:** 2016-05-09

**Authors:** Gi Seok Jeong, Da Yoon No, JaeSeo Lee, Junghyo Yoon, Seok Chung, Sang-Hoon Lee

**Affiliations:** 1 Department of Biomedical Engineering, College of Health Science, Korea University, Anam-ro 145, Seongbuk-gu, Seoul 02841, Korea; 2 Biomedical Engineering Research Center, Asan Institute for Life Sciences, Asan Medical Center, 88 olympic-Ro, Songpa-gu, Seoul 05505, Korea; 3 Department of Bioengineering, School of Engineering and Medicine, Stanford University, 443 Via Ortega, Stanford, California 94305, USA; 4 KU-KIST Graduate School of Converging Science and Technology, Korea University, Seoul 02841, Korea; 5 School of Mechanical Engineering, Korea University, Anam-ro 145, Seongbuk-gu, Seoul 02841, Korea

## Abstract

High-aspect ratio micro- and nano-structures have been used for the production of a variety of applications. In this paper, we describe a simple and cost-effective approach to fabricate an arrayed microarchitecture with an ultra-high aspect ratio using soft materials. The shapes and sizes of the honeycomb structure can be easily modulated by changing the dimensions and position of the base mould pattern and the pressure. The honeycomb structure is used to prepare a drug delivery patch and a microwell array to form cell spheroids without cell loss. The honeycomb structures prepared using natural ECM (collagen–Matrigel) materials are successfully fabricated. The hepatocytes and endothelial cells are seeded and co-cultured in the ECM-based micro-honeycomb to prepare a 3D liver model successfully mimicking an ultrastructure of liver and providing enhanced liver function.

## Abstract (web summary)

Tissue engineering applications call for controllable micro-structural units as a platform, but their fabrication remains challenging. Here, Jeong *et al*. show a method that enables soft materials to self-organize into highly packed micro-honeycomb structures with aspect ratios up to 500, and tunable shapes.

## Graphical abstract

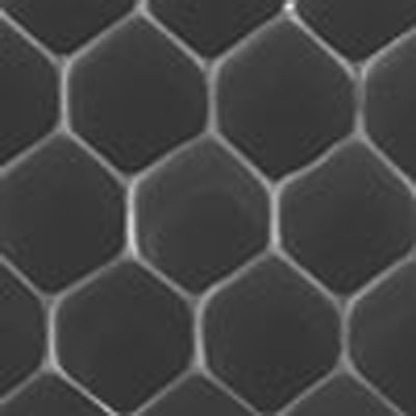

Recent progress in the field of micro and nanotechnologies has led to the production of a variety of complex micro- or nano-structures for use in sensors, actuators and battery applications^1^,^2^. Advanced micro- and nano-scale technologies have enabled broad applications of these structures, even in the biomedical fields of drug delivery, tissue engineering and medical devices^3^,^4^,^5^. Effective drug or cell delivery applications and tissue engineering techniques require structures that can pack a high number of cells^6^ or drugs as compactly as possible^7^. The construction of a structure with minimal dead space has received significant attention from scientists and engineers. One of the nature-inspired structures considered optimal for this purpose is the honeycomb structure prepared with a high aspect ratio (HAR) wall. For practical applications in the biomedical field, honeycomb structures must be formed from a soft material or a natural extracellular matrix (ECM). The fabrication of such honeycomb architectures using soft materials has presented a significant challenge due to the mechanical weakness inherent in HAR walls fabricated from soft materials. Conventional methods for fabricating HAR structures include deep reactive ion etching (D-RIE)^8^, lithographie–galvanoformung–abformung processes^9^ and UV lithography^10^,^11^. Although these approaches have provided ingenious solutions to fabricating diverse and challenging structures, they are low-throughput approaches, and the fabrication costs increase rapidly as the aspect ratio is increased. Some of these methods are limited to hard materials, and the construction of such structures using soft materials, such as poly(dimethylsiloxane) (PDMS) or ECM materials has been challenging^12^,^13^. The mechanical instabilities of natural ECM materials make it extremely difficult to build micro-honeycomb structures with ultra-thin walls^14^.

In this study, we propose a novel method of constructing self-organizing micro-honeycomb structure arrays consisting of ultra-high aspect ratio walls (with a maximum aspect ratio exceeding 500). The self-organization of the micro-honeycomb structures relies on the surface tension of the viscoelastic materials and the vacuum pressure. The shapes and dimensions could be readily controlled by changing the distance between the base mould patterns or by altering the vacuum pressure. Soft micro-honeycomb structures prepared from PDMS or natural ECM materials (collagen–Matrigel) were successfully fabricated. The PDMS structures displayed an excellent capacity for drug or cell loading. Hepatocyte and endothelial cells were seeded and co-cultured in the ECM hydrogel micro-honeycomb structures to fabricate a three-dimensional (3D) liver model consisting of compact cell spheroids and vessel-like structures that provided enhanced liver functions.

## Results

### Principles of viscoelastic lithography

**Figure 1:**
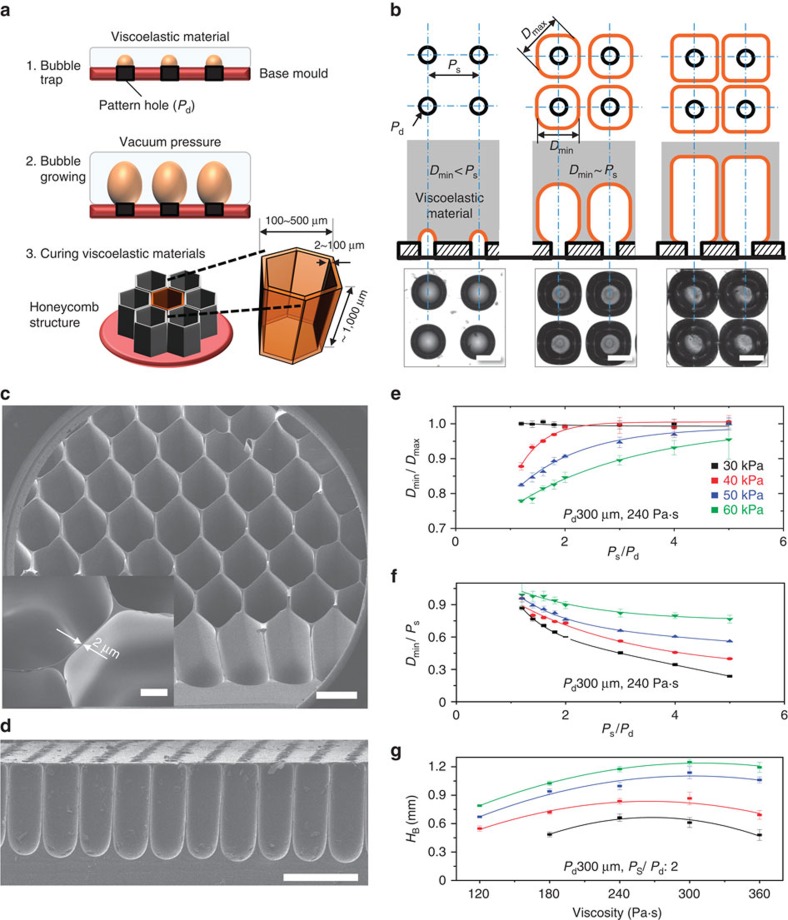
The principles underlying viscoelastic lithography. (**a**) The fabrication of tunable honeycomb microstructures using viscoelastic lithography. (**b**) Growth and interactions among bubbles in the HV-PDMS under a negative pressure (20 to 70 kPa below atmospheric pressure). The trapped air slowly increased in volume to form spherical bubbles in the HV-PDMS matrix (isotropic expansion phase). Polygonal bubbles were formed as the bubbles interacted with neighbouring bubbles (anisotropic expansion phase). The scale bar, 300 μm. (**c**) SEM image of the micro-honeycomb structures fabricated using viscoelastic lithography. The minimum wall thickness was 2 μm (inset, scale bar, 100 μm), and the aspect ratio was ∼500 (height to wall thickness). The scale bar, 500 μm. (**d**) Cross-sectional image of the micro-honeycomb structure. The scale bar, 500 μm. (**e**,**f**) The relationship between the polygonal factor *D*_min_/*D*_max_ and the pattern parameters over a range of pressures. Small separation distances between bubbles gave *D*_min_/*D*_max_ values approaching 1. (**g**) The height of the bubble as a function of the HV-PDMS properties over a range of pressure (30−60 kPa below atmospheric pressure). The bubble height in the HV-PDMS gradually increased, although it decreased under higher viscosity conditions (360 Pa·s). All error bars indicate the s.d.'s.

The principles underlying viscoelastic lithography are illustrated in Fig. 1a and Supplementary Fig. 1. A 10-mm-thick layer of a highly viscous PDMS (HV-PDMS) solution was poured onto a base mould with a patterned array of holes. The HV-PDMS solution did not permeate the holes in the array due to its viscosity. The air trapped in the array of holes increased in volume on application low pressures between −20 and −70 kPa, forming spherical bubbles in the HV-PDMS (isotropic expansion, Supplementary Fig. 1b). As the bubbles increase in volume, their shapes deformed in the presence of the growing neighbouring bubbles (anisotropic expansion, Supplementary Fig. 1c). Bubble growth in the viscoelastic materials depended mainly on three key parameters: the pressure, the size of each hole (*P*_d_), and the distance between the holes (*P*_S_). The formation of single-static bubbles under reduced pressure could be described using the Laplace equation^15^,^16^.

The Laplace equation for bubble formation is presented in equations (1, 2, 3),




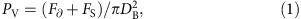




where




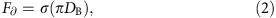







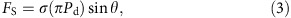




where *P*_V_ is the vacuum pressure, *σ* is the surface tension of the liquid, and *D*_B_ is the diameter of the bubble.

As shown in equations (1, 2, 3) and Supplementary Fig. 1a, the PDMS prepolymer required a high surface tension to balance the forces within the bubbles. However, conventional PDMS prepolymers have a low surface tension and an insufficient viscosity for constructing micro-honeycomb structures under a negative pressure. The PDMS prepolymer (using a 10:1 mixture of the PDMS prepolymer and the curing agent) viscosity was increased by thermally curing at 40 °C for 2 h, which raised the viscosity of the PDMS solution to ∼120 Pa·s. Shape-tunable micro-honeycomb structures were constructed using a two-phase expansion process, as shown in Fig. 1b and the Supplementary Fig. 1b,c. The bubbles in the HV-PDMS solution expanded isotropically as the pressure decreased, and the isotropic expansion was disrupted by interactions among neighbouring bubbles as the bubble size approached *P*_S_. The walls between growing bubbles became very thin and tall, forming tightly packed honeycomb structures. The schematic diagrams and optical microscopy images shown in Fig. 1b clearly illustrated this effect. Seen from the top, the bubble array formed a polygonal network as the bubbles expanded anisotropically, and the wall thicknesses between the bubbles decreased as the bubble depth increased. Top and side optical images acquired during the bubble growth process are shown in the Supplementary Fig. 1b. The bubble growth parameters could be controlled to produce micro-honeycomb structures with diverse shapes. A scanning electron microscopy (SEM) image of a micro-honeycomb structure is shown in Fig. 1c. The minimum wall thickness was 2 μm, as shown in the inset in Fig. 1c, and the structure had a maximum aspect ratio of ∼500. The proposed method is much cheaper and requires much less time than other methods^8^,^17^. During the fabrication process, PDMS underwent three status transitions, from a liquid (low viscoelastic property), to a viscoelastic liquid (highly viscoelastic), to a solid. The bubble growth process was performed during the viscoelastic stage. Diverse bubble shapes were produced using different combinations of three parameters: (1) the pattern parameters (*P*_S_ and *P*_d_), (2) the material parameters (the viscoelastic properties of the PDMS), and (3) the force parameter (pressure). *P*_S_, *P*_d_ and the width of a bubble are defined in Supplementary Fig. 2. During isotropic expansion, the polygonal factor (*D*_min_/*D*_max_, where *D*_min_ is the horizontal diameter of a polygonized bubble, *D*_max_ is the diagonal diameter of a polygonized bubble) is defined as illustrated in Supplementary Fig. 2b. A polygonal factor of ‘1' produces bubble growth during isotropic expansion. Supplementary Fig. 3 shows the bubble shape change as a function of *P*_S_ and the pressure. During expansion, the bubbles underwent isotropic and anisotropic expansion, finally reaching equilibrium. The effects of the pattern and force parameters on the polygonal factor were measured and are illustrated in Fig. 1e,f. These graphs reveal that the shape of a bubble becomes polygonal at lower *P*_S_ values and higher negative pressures. Figure 1g shows the height of a bubble as a function of the viscosity over a range of pressures (30–60 kPa below atmospheric pressure). The height of the bubble gradually increased on application of increasingly negative pressures or increasing viscosity, whereas the bubble height decreased at a viscosity of 360 Pa·s.

### Modulation of the micro-honeycomb structure shape

**Figure 2:**
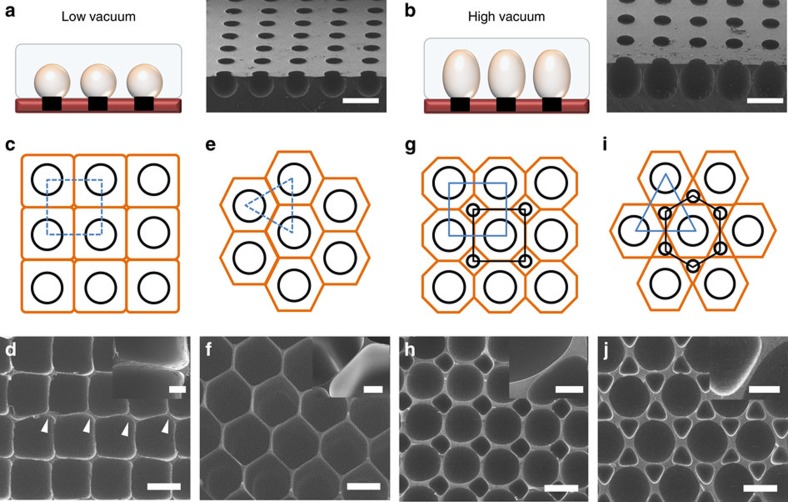
Modulation of micro-honeycomb structures. (**a**,**b**) The same base mould was used to tune the growing bubble patterns by controlling the vacuum pressure. (**a**) During the initial stage, the growing bubbles were spherical in shape. (**b**) Under high vacuum conditions, the growing hemispherical bubbles formed a jar-like structure. (**c**) A gridiron-like (blue dotted line and black circles) hole arrangement produced cuboidal full-sized bubbles (orange rectangles). (**d**) SEM image of the cuboidal structure formed. The minimum wall thickness between cuboids was <2 μm (scale bar in the inset: 20 μm). Some misalignment events (white arrowheads) were present, indicating that unstable forces were generated as the bubbles grew. (**e**,**f**) A triangular (blue dotted lines) hole-pattern (black circles) generated a well-organized uniform hexagonal structure with a HAR. (**f**) SEM image of a hexagonal bubble pattern. The inset shows that the wall thickness was <2 μm (scale bar in the inset: 20 μm). (**g**,**h**) A pattern formed from holes of two sizes: 300 and 100 μm. (**g**) The 300 and 100 μm holes were arranged in a rectangular pattern (blue and black dotted lines). A large octagon surrounded by four small rhombus shapes was created (orange line). The small rhombus-shaped bubbles exerted supportive forces on the large octagonal bubbles. (**h**) SEM image of the large octagonal and small rhombus-shaped structures. The walls were ∼5-μm-thick, as shown in the inset, thicker than the walls of the rectangular and hexagonal structures (scale bar in the inset: 50 μm). (**i**) The 300 and 100 μm holes were arranged in triangular and hexagonal patterns, respectively (indicated by the blue and black dotted lines). A large hexagonal bubble surrounded by six small triangular bubbles was created as the bubbles expanded. (**j**) SEM image of the large hexagonal structures surrounded by six small triangular structures (scale bar in the inset: 50 μm). Scale bars in **a**,**b**,**d**,**f**,**h** and **j** indicate 400 μm.

Figure 2 shows the diverse micro-honeycomb structure shapes that resulted from various parameter combinations. The base mould was held constant, and the growing bubble patterns were changed by controlling the vacuum pressure (Fig. 2a,b). Under low vacuum conditions (30 kPa), the growing bubbles formed spherical shapes (Fig. 2a). Intermediate vacuum conditions (50 kPa) could be used to tune the growing hemispherical bubbles to form a jar-like structure (Fig. 2b). High vacuum conditions (>60 kPa) created a densely packed honeycomb structure. The locations and sizes of the hole-pattern could be adjusted to modulate the shape and size of the honeycomb structures. Densely packed polygonal structures with different spacings and arrangements could be produced using a single hole-pattern, as shown in Fig. 2c–j. A gridiron-like pattern of holes (Fig. 2c, black circles) produced cuboid structures with square cells when viewed from the top (Fig. 2c, orange line). An SEM image of the cuboidal structure is shown in Fig. 2d. The minimum wall thickness between cuboid cells was <2 μm (Fig. 2d, inset), and the structure was ∼1 mm high. A few instances of misalignment among the cuboid wells were observed (indicated by white arrowheads in Fig. 2d), possibly caused by imbalances among the forces operating at the position at which the four bubbles met. By contrast, a triangular pattern (indicated by the blue dotted line in Fig. 2e) created a well-organized and uniform hexagonal structure with a HAR (Fig. 2e, orange line). The SEM image of the hexagonal bubble pattern formed readily reveals the honeycomb structure, as shown in Fig. 2f. Unlike the cuboidal structures, the hexagonal structures were completely aligned and no deformations were found. These results suggested that the forces were balanced and well regulated at the three-bubble intersections in the hexagonal structures. Although the structures were 1 mm high, the wall thickness was <2 μm (Fig. 2f, inset) due to the stable balance of forces between the growing bubbles. Hole-patterns composed of holes with different sizes (300 and 100 μm) were used to fabricate structures consisting of a heterogeneous honeycomb shape by appropriately positioning the holes. Positioning the 300 and 100 μm holes in a rectangular array (illustrated by the blue and black dotted lines in Fig. 2e) led to the formation of large octagonal bubbles, each of which was surrounded by four small rhombus-shaped bubbles (Fig. 2h, orange line). The small rhombus-shaped bubbles exerted supportive forces on the large octagonal bubbles. An SEM image of the resulting large octagonal and small rhombus-shaped bubble is shown in Fig. 2h. Unlike the rectangular bubbles (Fig. 2c,d) described above, larger stable bubbles could be grown using the heterogeneous structure because of the support provided by the small rhombus-shaped bubbles. In other words, the smaller bubbles present between the larger bubbles stabilized the forces as the bubbles grew. The wall of this structure was ∼5-μm-thick (thicker than the walls formed in the cuboid and hexagonal structures), as shown in the inset of Fig. 2h. Arranging the 300 and 100 μm holes in triangular and hexagonal patterns (illustrated with blue and black dotted lines in Fig. 2i), respectively, permitted the formation of large hexagonal bubbles, each of which was surrounded by six small triangular bubbles, as the bubbles expanded (Fig. 2j, orange line). An SEM image of the large hexagonal bubbles surrounded by six small triangular bubbles is shown in Fig. 2j. These heterogeneous micro-honeycomb structures were uniform in shape, and the walls were ∼10-μm-thick, as shown in the inset of Fig. 2j. These results indicated that heterogeneous and densely packed micro-honeycomb structures could be produced simply by altering the positions and sizes of the hole-pattern. Additional useful structures could potentially be produced using appropriate patterns and designs to optimize the bubble expansion process.

### Applications of the micro-honeycomb structures

**Figure 3:**
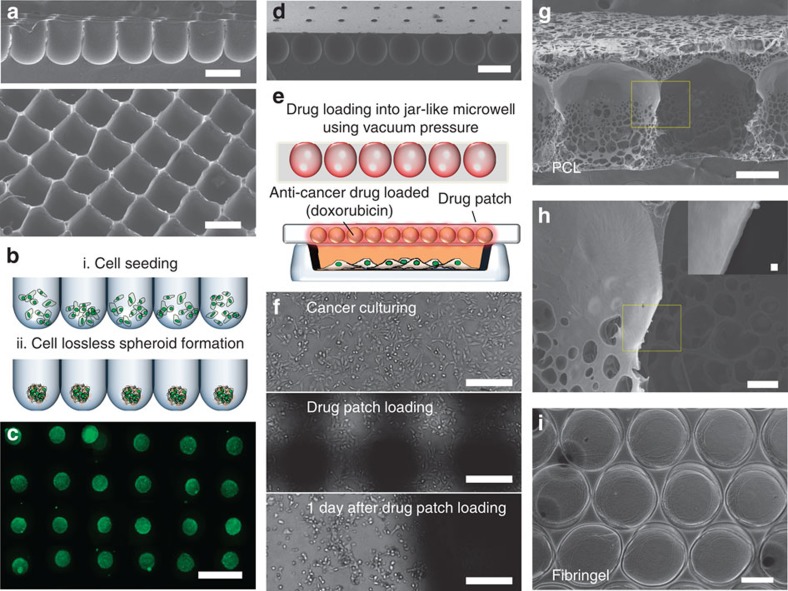
Diverse applications of the tunable HAR honeycomb microstructures. (**a**) SEM images of the rectangular microstructures for lossless cell seeding. (**b**) Schematic diagram of the lossless cell seeding process. (**c**) Mouse embryonic stem cells (mESCs) were seeded into the HAR microstructure without the need for additional washing processes. Three days after seeding, the mESCs self-aggregated and formed spheroids in the HAR microwell. The spheroids are shown after staining using a live/dead assay. (**d**) Drug delivery patch using the jar-like microstructure array. (**e**) Schematic diagram of drug loading into the jar-like HAR honeycomb microstructure. (**f**) Breast cancer cells (MCF-7) were cultured on the 35-mm culture dish. The drug-loaded (1% doxorubicin hydrochloride, Sigma-Aldrich, MO, USA) patch was applied to cover the dish. One day after drug patch loading, most MCF-7 cells in the dish were dead. (**g**,**h**) polycaprolactone (PCL)-based HAR honeycomb microstructure with a wall thickness on the submicron scale (inset, scale bar, 1 μm). (**i**) Fabrication of the fibrin gel-based HAR honeycomb microstructure. (The scale bar indicates 300 μm.) The scale bars in **a**,**c** and **d** indicate 500 μm, **f** and **g** indicate 200 μm and **h** indicates 30 μm.

Figure 3 illustrates several applications of the micro-honeycomb structures. Cell spheroid formation plays an important role in tissue engineering^18^,^19^, drug screening^20^ and stem cell research^21^,^22^. During cell spheroid formation in a microwell array, lossless cell seeding and harvesting of intact cell spheroids is important^22^. The use of thin microwell array walls enables the lossless seeding. Cell losses in various concave PDMS microwell arrays with different inter-wall distances were measured, and the results are presented in Supplementary Fig. 4. Comparing to theses microwell arrays, the rectangular microstructure allowed the lossless cell seeding and the washing steps to remove undocked cells were not needed (Fig. 3a,b). Spheroid formation was achieved using mouse embryonic stem cells. Three days after seeding, the mouse embryonic stem cells self-aggregated and formed spheroids, as shown in Fig. 3c. A fluorescent image of the live/dead assay (green: live, red: dead) revealed that most cells were alive in the microwell culture array (Fig. 3c). These spheroids could be used as embryoid bodies to induce stem cell differentiation.

In another application, we fabricated jar-like micro-honeycomb structures for use as drug-releasing patches (Fig. 3d,e). A patch comprising the jar-like structures loaded with an anticancer drug (10 μg ml^−1^ doxorubicin hydrochloride) was placed on a cell culture dish. The anticancer drug (doxorubicin) diffused out from the drug patch, and 1 day after drug patch application, most MCF-7 cells in the dish detached from the surface (Fig. 3f). A variety of viscoelastic materials could be used for the viscoelastic lithography. We fabricated 10% (w/v) polycaprolactone micro-honeycomb structures with a wall thickness on the submicron scale (Fig. 3g,h). The inset shown in Fig. 3h reveals the submicron wall thickness. A mixture of fibrinogen and thrombin were also used to fabricate ECM micro-honeycomb structures (Fig. 3i).

### 3D liver model in the micro-honeycomb structure

**Figure 4:**
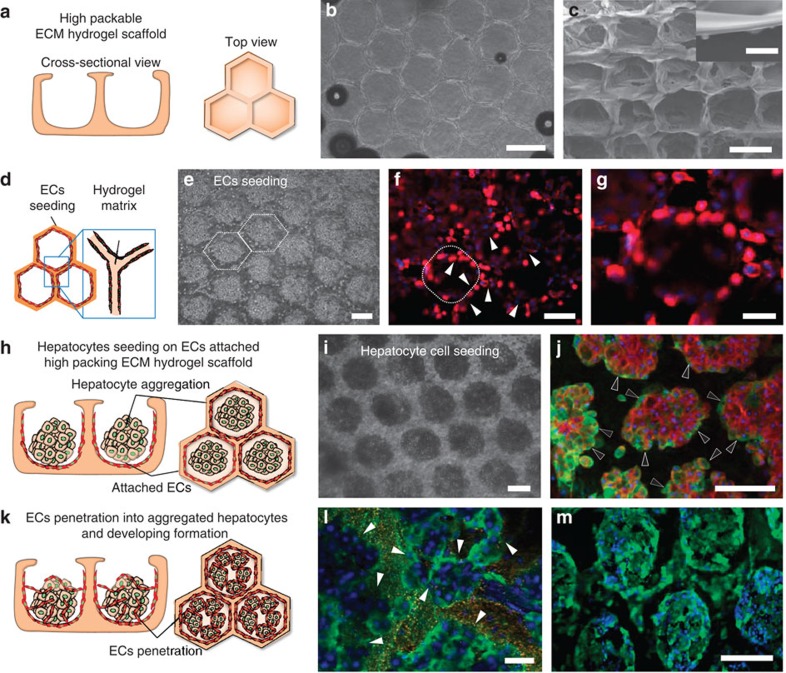
3D-tunable micro-honeycomb structure of an ECM matrix for use as a co-culturing system. (**a**) Schematic diagram of the ECM matrix used as a co-culturing system. (**b**) Optical microscopy image of the patterned collagen–Matrigel (ratio of 7:3) matrix. (**c**) SEM image of the patterned Matrigel micro-honeycomb structure (aspect ratio of 400). The inset shows the sidewall thickness (2 μm) produced at a pressure 70 kPa below atmospheric pressure (scale bar, 10 μm). (**d**) Schematic diagram of the endothelial cells attached to the patterned hydrogel matrix. (**e**) Optical microscopy image collected after endothelial cell seeding. (**f**) Endothelial cells were attached to both sides of the cuboidal patterned hydrogel matrix sidewalls. Red and blue indicate CD31 and DAPI, respectively. (**g**) Magnified view of **f**. (**h**) Schematic diagram of the hepatocyte seeding process and cell aggregation on the endothelial cell-patterned hydrogel. (**i**) Optical microscopy image after hepatocyte seeding. (**j**) Immunostaining of the serum albumin (green, black arrowheads) and actin (red), revealing that albumin was secreted by the patterned hepatocytes. (**k**) Schematic diagram of the endothelial cells that penetrated the hepatocyte aggregates and void structure. (**l**) Immunostaining of the serum albumin (green) revealed that albumin was secreted into the void structures of the hepatocytes (white arrowheads; scale bar, 10 μm). (**m**) The void structures of the hepatocytes displayed high levels of CYP450 activity (green). The scale bars in **b**,**c**,**e**,**f**,**g**,**i**,**j** and **m** indicate 500 μm.

**Figure 5:**
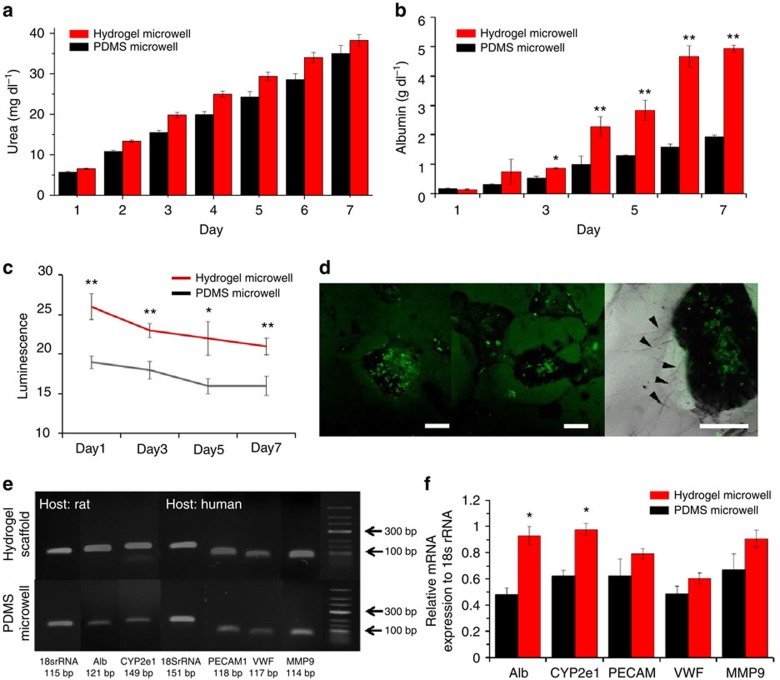
Functional assays of the co-cultured spheroids in a 3D hydrogel scaffold and PDMS microwells. (**a**) Urea secretion test. No significant differences were observed between the PDMS microwells and the hydrogel microwells. (**b**) Albumin secretion test. The hydrogel microwell group displayed significantly higher levels of albumin secretion after day 3. (**c**) CYP3A4 enzymatic activity test. The *y* axis corresponds to the luminescence level. (**d**) Confocal images of fluorescein diacetate staining. The accumulated green fluorescence demonstrates bile duct formation. The black arrows indicate proliferated endothelial cells that formed vessel-like structures (scale bars, 200 μm). (**e**) Gene expression of the co-cultured spheroids and quantified results of mRNA gene PCR based on 18s rRNA expressions. (**f**) Alb and CYP2E1 displayed significantly higher expression levels in the 3D matrix group (*P<0.05, **P<0.01). All error bars indicate the s.d.

A proof-of-concept tissue engineering application of the scaffold was demonstrated by seeding hepatocytes and Endothelial cells in the natural ECM micro-honeycomb structure to fabricate a 3D liver-like tissue (Fig. 4a). We used an ECM (a 7:3 mixture of collagen and Matrigel) honeycomb structure as a scaffold to pack as many cells as possible into a given volume (Fig. 4b). An SEM image of the natural ECM honeycomb structure (with an aspect ratio of *ca.* 400) is shown in Fig. 4c. The sidewall was ∼2-μm-thick (Fig. 4c, inset). Before hepatocyte seeding, endothelial cells were seeded (Fig. 4d) and permitted to grown uniformly over the scaffold (Fig. 4e, day 0) across the bottom and sidewalls of each well (as indicated by the white arrowheads in Fig. 4f,g, day 1). Primary hepatocytes harvested from rat liver were seeded and cultured (Fig. 4h,i) and inoculated onto the hydrogel scaffolds coated with endothelial cells. The hepatocytes aggregated very quickly and displayed excellent albumin expression, as indicated by the black arrowheads in Fig. 4i,j. Figure 4k shows a schematic diagram of the EC penetration into the hepatocyte aggregates. Supplementary Fig. 5g presents a confocal image of a spheroid cross-section, revealing void structures within the hepatocyte aggregates. Some endothelial cells were present in the void structures and penetrated into the hepatic aggregates of the scaffold. The liver functions of this engineered tissue were characterized by analysing the serum albumin (Fig. 4j,l) expression and CYP450 (Fig. 4m) activity using immunofluorescence staining. As shown in Fig. 4j, the hepatic structures expressed serum albumin actively (green and black arrowheads). Figure 5a,b show that the hepatocyte-EC co-culture models in the hydrogel microwell displayed higher levels of albumin and urea secretion than do conventional spheroid culturing models over a period of 1 week. Albumin secretion in the hydrogel microwell group was 2.55 times higher than the value obtained from spheroid co-culturing models in PDMS microwells (Fig. 5b). As shown in Fig. 5c, the activity of CYP3A4 the hydrogel microwell group over 1 week was 131% of the value obtained from the spheroids prepared using PDMS microwells. Confocal images of fluorescein diacetate accumulation suggested that bile duct formation occurred in the spheroids prepared in the hydrogel microwells (Fig. 5d, left). The black arrows indicate the proliferating endothelial cells that formed vessel-like structures (Fig. 5d, right). Prevascularization of the engineered tissues with a controlled 3D structure is advantageous for implantation and engraftment. The hydrogel microwell played a huge role in inducing the formation of these structures and endothelial cells are more viable and able to preserve their specific functions in a natural hydrogel. The enhanced hepatic function and endothelial characteristics were evaluated by conducting reverse transcription PCR. As shown in Fig. 5e, the expression of ALB and CYP2E1, a member of the cytochrome P450 family, were significantly higher in the hydrogel microwell group than in the control group. We analysed the genes related to endothelial and proangiogenic characteristics, such as PECAM1, vWF and MMP9; however, no significant differences between the two culture methods were observed (Fig. 5e). Although EC-related gene expression also showed no significant differences between the two culture methods, the morphological features of the endothelial cells were quite different. Co-cultured spheroids in the PDMS microwells did not reveal proliferating endothelial cells on the PDMS surface (Supplementary Figs 6 and 9), whereas the endothelial cells in the hydrogel microwell actively proliferated. Seeding the hepatocytes in the EC pre-seeded hydrogel microwell resulted in the formation of hepatocyte aggregates. A 3D liver model with vessel-like structures around the spheroids and on the hydrogel matrix appeared on day 5 (Fig. 5d). Similar amounts of albumin were secreted by both models between days 1 and 3; however, the co-culture model expressed more albumin from day 4 onwards compared with the mono-culture model. The differences between the amounts of albumin secreted by the two models increased as time passed (Supplementary Fig. 5h,i). The albumin and urea secretion rate per cell are described in the Supplementary Note 1. These results were further supported by results obtained in previous studies, in which co-cultured endothelial cells were found to assist hepatocytes in maintaining their features *in vitro* over an extended period of time^23^,^24^,^25^,^26^.

## Discussion

Recent rapid progress in material and microfabrication technologies enables the development of a variety of soft structures, including 3D scaffolds for tissue engineering applications. Despite such progress, the construction of 3D soft structures with HAR thin wall has presented a significant challenge. The novel self-organizing method proposed here facilitates the laboratory-based construction of a variety of soft micro-honeycomb structure arrays with ultra-thin walls without the use of complicated or expensive processes and facilities. The sizes and shapes of the HAR structures could be modulated simply by changing the base mould pattern and applied vacuum pressure. The fabricated structures were used to demonstrate a variety of applications, including uniform cell spheroid formation and drug delivery patches. With the proposed method, we demonstrated the preparation of a 3D HAR scaffold composed of a very soft hydrogel, including collagen. Such scaffolds are beneficial in providing an *in vivo*-like environment. Although recent progress in technology has enabled the development of biological substitutes that can restore, maintain or improve the functions of tissues or whole organs, the scale-up and creation of physiologically relevant tissues with well-distributed vascular systems continues to pose a challenge. The development of highly organized functional tissue-level organoids requires spatially controlled co-cultures of heterogeneous cell types. Various patterned hydrogel scaffolds have been developed using photo-patterning^27^,^28^, microscale stamping^29^, lift-off^2^ techniques and 3D printing; however, it is difficult to construct patterned hydrogel scaffolds that can support the development of complex native ultrastructures while maintaining organ-specific function. The proposed method allows the construction of HAR honeycomb structures using a soft hydrogel without the need for demoulding processes, followed by the successful culture of heterogeneous cells. As shown in Supplementary Fig. 5a,b, residual stress at the solid–solid interface between the base mould and the natural ECM hydrogel material was not observed, indicating that large-area densely packed micro-honeycomb structures could be easily constructed (Supplementary Fig. 5c–e). We successfully cultured a 3D liver structure consisting of hepatocytes and endothelial cells. The co-cultured hepatocytes and endothelial cells displayed high levels of albumin and urea secretion over the course of 1 week, in addition to excellent cell–cell interactions. These results indicated that the hydrogel microwell provided a suitable environment for engineering liver tissues consisting of compactly packed cells (>10^10^ cells) and vascular structures.

In summary, we successfully produced shape-tunable densely packed soft micro-honeycomb structures using only the viscoelastic properties of a material. The PDMS and natural ECM structures described here are, to the best of our knowledge, the first honeycomb structures with ultra-HAR walls reported to date. The shapes and sizes of the structures could be adjusted by controlling the applied pressure and the distance between bubbles, as characterized experimentally. Natural ECM hydrogel micro-honeycomb structures will be extensively applied towards the fabrication of engineered 3D tissues for failed organ function regeneration. The tunable pattern size, shape and the possibility of mass-producing the structures are critical advantages for the construction of a range of structures outside of the biomedical field that require a high-packing density, for examples, devices that require high-void densities, such as batteries.

## Methods

### Preparation of HV-materials

The HV-PDMS was prepared by pre-heating the PDMS prepolymer (a mixture of PDMS and the curing agent in a ratio of 10:1) for 120 min at 40 °C. The viscosity of the PDMS prepolymer was critical and was monitored using a rotational viscometer (HBDV-II; Brookfield Engineering, Middleboro, MA, USA, Supplementary Fig. 7). A 5-mm-thick layer of the HV-PDMS solution was poured onto a base mould with an appropriate pattern of holes, and the HV-PDMS did not penetrate the holes. The polycaprolactone honeycomb microstructure was fabricated by preparing polycaprolactone (10% w/v, Sigma, St Louis, MO, USA, dissolved in dimethylformamide/tetrahydrofuran). The pressure was decreased to a value between 20 and 70 kPa below atmospheric pressure, and the air trapped in the array of small holes increased in volume to form spherical bubbles in the HV-PDMS (Supplementary Fig. 8).

### Natural ECM hydrogel patterning

The collagen–Matrigel matrix was fabricated from Matrigel and type 1 collagen purchased from BD Bioscience (San Jose, CA, USA). The gel stiffness could be varied by varying the pH of the pre-polymerized collagen solution over the range pH 7.4–11 (ref. 30). In this study, a collagen solution with an *in vivo*-like pH of 7.4 was used to prepare the collagen–Matrigel matrix polymerization process. The collagen was diluted in sterile water, and the pH was adjusted by adding a 10 × phosphate-buffered saline (PBS) solution containing phenol red and 0.5 N NaOH. Once the pH-regulated collagen had been prepared, the Matrigel and collagen were mixed in a collagen:Matrigel ratio of 7:3. A desiccator was pre-heated to 40 °C, the collagen–Matrigel mixture was gently poured onto a patterned PDMS plate in the desiccator, and a vacuum was applied to the mixture for 30 min to allow the required patterns to form in the collagen–Matrigel mixture (Supplementary Fig. 5). The fibrin honeycomb microstructure was fabricated using a 1:1 fibrin:thrombin mixture (Sigma-Aldrich, St Louis, MO, USA) poured onto the base mould within 1 min of mixing.

### Preparation of cells and culture

The human umbilical vein endothelial cells (HUVECs) were purchased from Lonza (Basel, Switzerland) and were cultured in an endothelial cell growth medium (ScienCell Research Laboratories, Carlsbad, CA, USA) containing 5% foetal bovine serum, 20 ng ml^−1^ vascular endothelial cell growth factor and 1% penicillin/streptomycin. The HUVECs were cultured for no more than seven passages. Primary hepatocytes were isolated from 8-week-old male Sprague–Dawley rats (DBL, Seoul, South Korea) using a two-step collagenase perfusion procedure. The cell viability was determined using the trypan blue exclusion method. The isolated cells were >90% viable and were used in subsequent experiments. The hepatocytes were cultured in high-glucose Dulbecco's modified Eagle's medium supplemented with 20 mM HEPES (Sigma-Aldrich, St Louis, MO, USA), 25 mM NaHCO_3_, 30 mg ml 21 L-proline, 10% foetal bovine serum, 25 U ml^−1^ penicillin, 25 μg ml^−1^ streptomycin, 10 μg ml^−1^ gentamicin, 10 ng ml^−1^ epidermal growth factor, 50 ng ml^−1^ insulin, 10^−4^ M dexamethasone, 10 mM nicotinamide and 100 mM L-ascorbic acid.

A 100-μl aliquot of a HUVEC suspension (5 × 10^4^ cells ml^−1^) was seeded onto the patterned hydrogel, and the device was placed in an incubator at 37 °C for 4 h to allow the cells to become attached to the hydrogel matrix. A 500-μl aliquot of the primary hepatocyte suspension (2 × 10^5^ cells ml^−1^) was then seeded onto the HUVEC—hydrogel matrix. The matrix was then kept at 37 °C in an incubator (with a 5% CO_2_ atmosphere) to culture the cells. Cell responses were monitored daily using phase contrast (AMG; Westover Scientific, Bothell, WA, USA) and confocal microscopy (Olympus, Tokyo, Japan). The HUVECs mixture and the hepatocyte medium were replaced every other day during the cell culture process. All animal procedures were conducted according to the Korean University IRB guidelines and after approval by the appropriate institutional review committees.

### Albumin and urea analyses

Albumin and urea secretion were analysed by measuring the albumin and urea concentrations in a medium conditioned by the cultured models (Supplementary Note 1). Patterned cell sheets were cultured in 48 wells over 7 days. A 500-μl aliquot of the medium was removed at each of the chosen time points for the assay, and this volume was replaced with 500 μl of fresh medium.

### CYP450 luminescence assay and fluorescein diacetate staining

Cytochrome P450 3A4 (CYP3A4) enzymatic activity was measured using the luminescent P450-Glo CYP3A4 Cell-based Assay (Luciferin-PFBE, Promega), as described by the manufacturer. Briefly, cells were incubated with a luminogenic substrate (Luciferin-PFBE) in a culture medium for 4 h at 37 °C. The medium was subsequently transferred to a 96-well opaque white plate, and an equal volume of Luciferin Detection Reagent was added to initiate a luminescent reaction. The plate was incubated at room temperature for 20 min and luminescence was read using a Multimode Plate Reader (PerkinElmer). The spheroids were stained with fluorescein diacetate to reveal bile duct formation. Spheroids were incubated in a culture medium containing 2.5 μg ml^−1^ fluorescein diacetate (Sigma) for 40 min in an incubator. After 40 min, the medium was replaced, and the spheroids were incubated in the absence of fluorescein diacetate for 40 min. The fluorescein distribution over the spheroids was observed using confocal microscopy (Olympus, Tokyo, Japan).

### Immunostaining

Endothelial and primary hepatocyte cells were fixed using 4% PFA by incubating them at 4 °C for 30 min in 0.1% Triton X-100, then incubating them in PBS for 20 min at room temperature. The cells were rinsed with 0.1% BSA in PBS, incubated with Block Ace (Dainippon Pharma, Tokyo, Japan) at 4 °C for 30 min, and then incubated with CD31 antibody (Abcam, Cambridge, UK), cytochrome P450 reductase antibodies (Abcam, Cambridge, UK) and serum albumin (Santa Cruz Biotechnology Inc., Santa Cruz, CA) overnight at 4 °C. The cells were then rinsed with 0.1% BSA in PBS and incubated with Alexa Fluor 488-conjugated anti-rabbit IgG secondary antibodies at 4 °C for 90 min. The cells were incubated with 4,6-diamidino-2-phenylindole for 5 min at room temperature.

### Gene analysis

After 9 days of culturing, co-cultured spheroids composed of hepatocytes and Endothelial cells were retrieved from the PDMS microwells and from the 3D ECM scaffold after collagenase treatment for 12 h (Collagenase type 1, 1 mg ml^−1^). RNA was purified using the RNeasy kit (Qiagen, CA, USA) and cDNA was generated using reverse transcriptase (TAKARA, Japan) according to the manufacturer's instructions. The primer sequences are listed in the Supplementary Table 1.

## Additional information

**How to cite this article:** Jeong, G. S. *et al*. Viscoelastic lithography for fabricating self-organizing soft micro-honeycomb structures with ultra-high aspect ratios. *Nat. Commun.* 7:11269 doi: 10.1038/ncomms11269 (2016).

## Supplementary Material

Supplementary Information Supplementary Figures 1-9, Supplementary Table 1, Supplementary Note 1 and Supplementary Reference
