# Supplementary material for: Viscoelastic lithography for fabricating self-organizing soft micro-honeycomb structures with ultra-high aspect ratios
Source: Nat Commun. 2016 May 9;7:11269. doi: 10.1038/ncomms11269 (PMC4865738; doi:10.1038/ncomms11269)
Supplement: Supplementary Information — Supplementary Figures 1-9, Supplementary Table 1, Supplementary Note 1 and Supplementary Reference [file ncomms11269-s1.pdf]

Supplementary information

Supplementary Figures

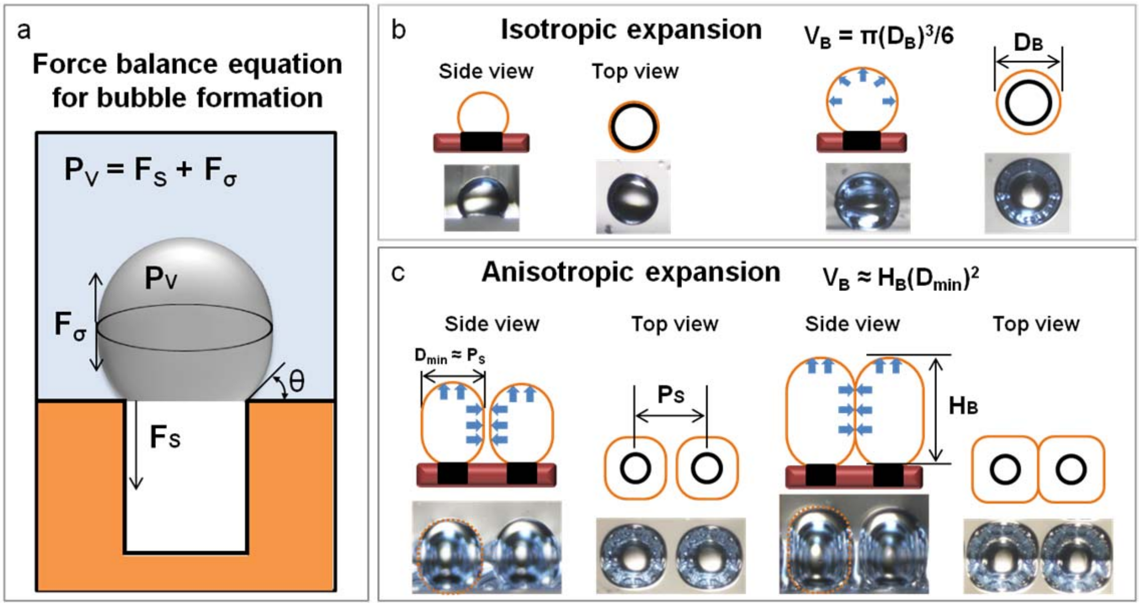

Supplementary Figure1. Schematic of the bubble growth process. (a) Schematic of the force balance required for bubbles to form. Applying a vacuum causes a bubble to grow in a way that depends on the surface tension of the bubble and hole surface. (b) Isotropic expansion of a bubble. (c) Anisotropic expansion of a bubble. The bubbles grow into polygonal structures because of the interfering forces exerted by neighbouring bubbles.

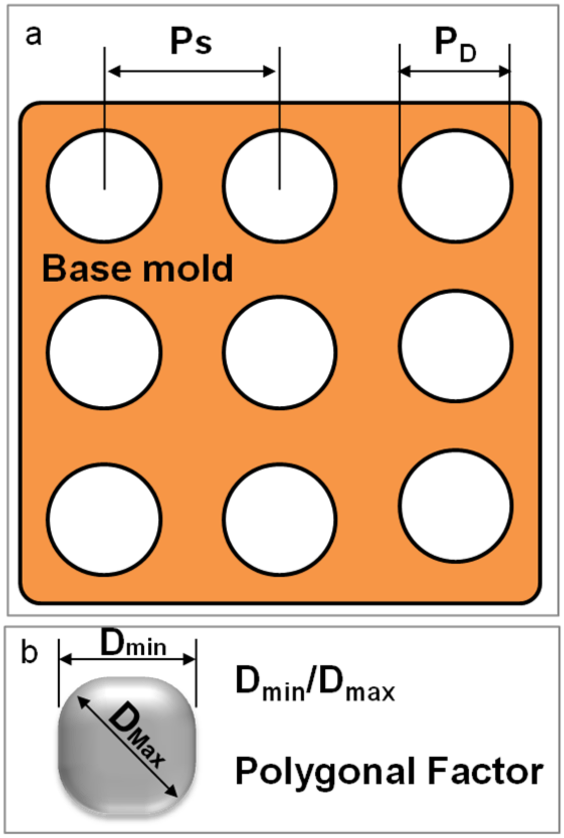

Supplementary Figure2. Schematic showing the notation used. (a) Notation for the base mould. (b) Definition of the polygonized factor (D_{min} /D_{max}). D_{max} is the diagonal diameter of the bubble.

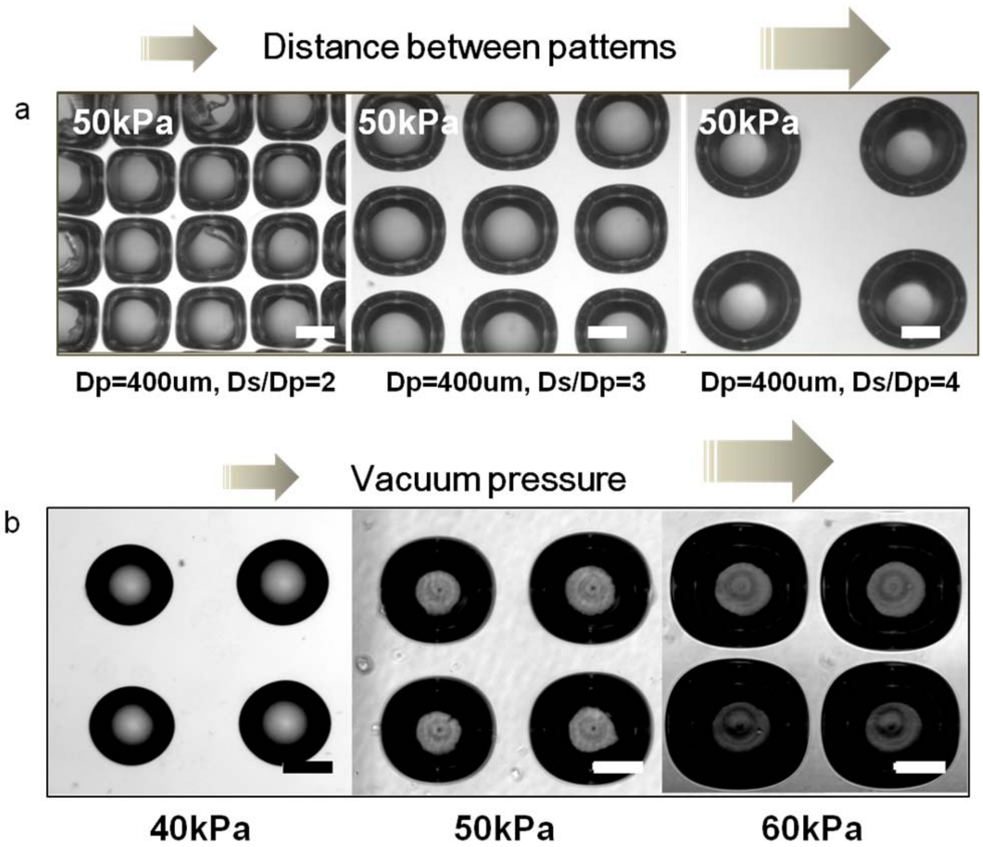

Supplementary Figure3. Relationship between the vacuum and the distance between the holes. (a) At a fixed vacuum (50 kPa below atmospheric pressure), changing the P_S affects the polygonization of the bubbles. (c) At a fixed distance between the holes in the base mould, changing the pressure affects the polygonization of the bubbles.

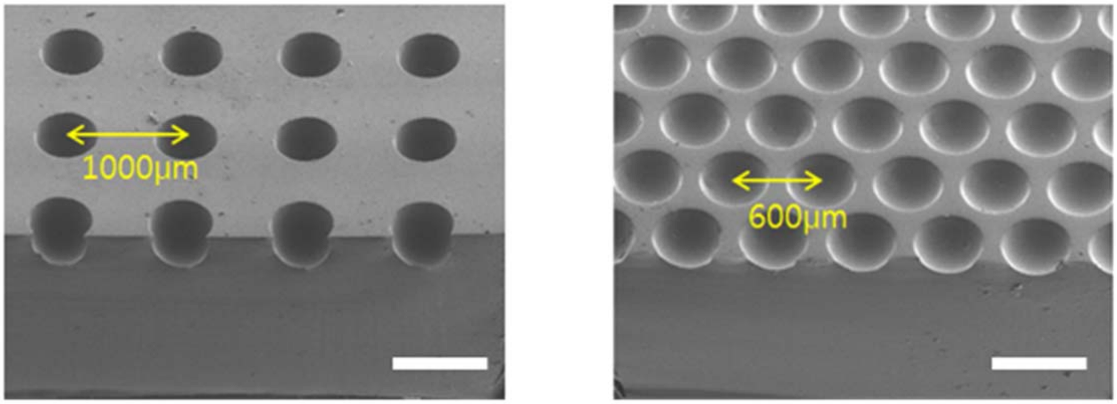

Well type (D=500µm) (Distance between wells)	Cell loss rate (%) N=4
Concave microwell (1000 µm)	65.3 ± 5.2
Concave microwell (600 µm)	26 ± 3.2
Microhoneycomb	< 1%

Supplementary Figure4. SEM image of concave wells with different distance between wells and cell loss rate. Scale bars indicate 500 µm.

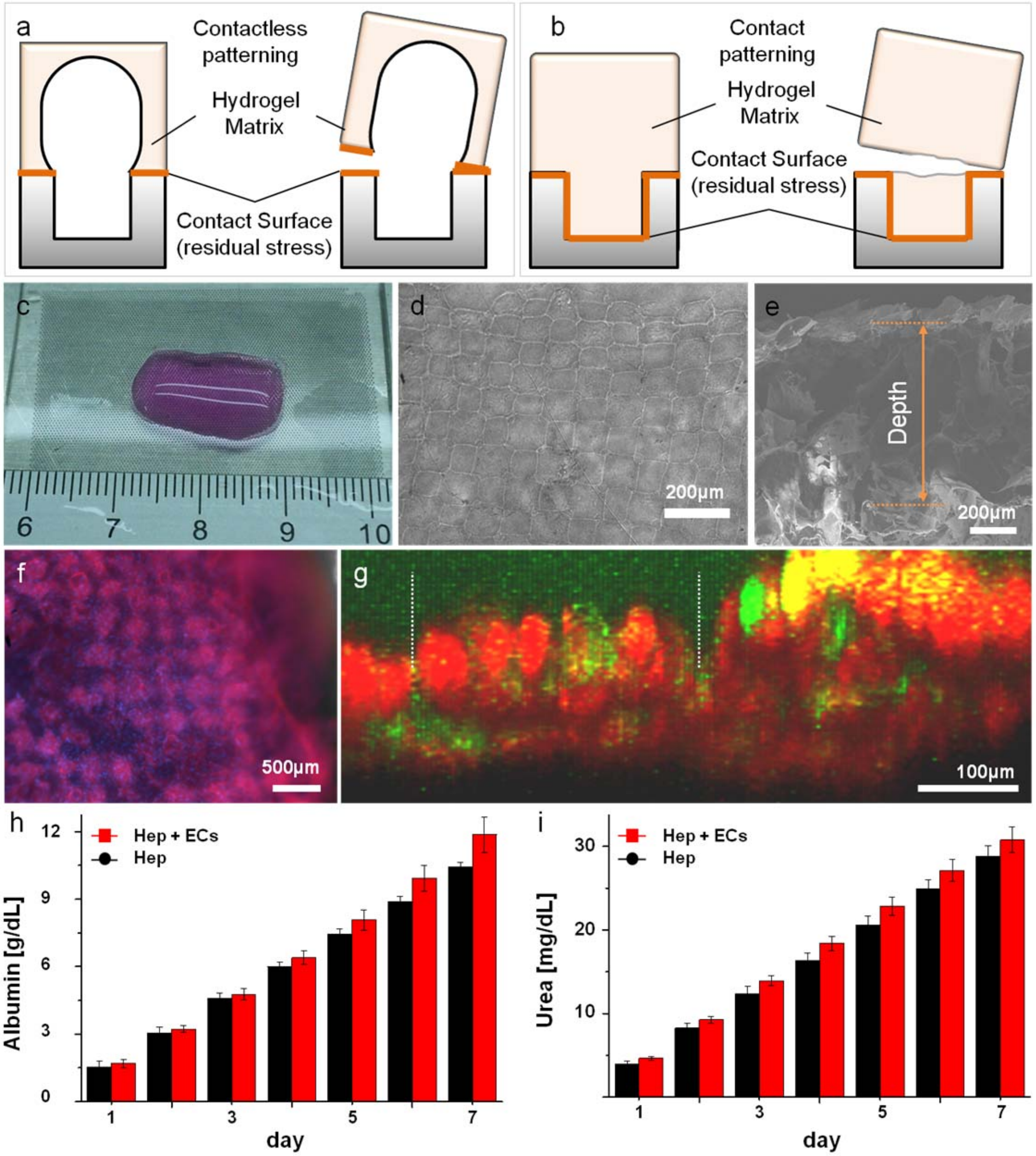

Supplementary Figure5. Creating a patterned hydrogel matrix using growing bubbles. (a) Solid–solid contactless hydrogel printing using growing bubbles allows the residual stress at the hydrogel–base mould surface to be minimized. (b) Conventional solid–solid contact hydrogel printing, in which the hydrogel matrix is easily torn because of the residual stress at the hydrogel–base mould surface. (c) A hydrogel (collagen–Matrigel) on a scale of a few centimetres patterned using growing bubbles. (d) An optical microscopy image of the patterned collagen hydrogel (aspect ratio: about 300). (e) SEM image of the side of the Matrigel, the Matrigel is about 800 μm deep. (f) A fluorescent microscopy image of a cuboid-patterned collagen–Matrigel matrix (aspect

ratio: about 400). (g) Cross sectional confocal microscope image of the patterned hepatocyte and ECs. Immunostaining of the serum albumin (green and black arrowheads) and actin (red). White dotted line indicates the boundary of the ECM scaffold. White dotted lines indicate the boundary of the natural ECM scaffold. (h–i) Both cell culture models (hepatocyte only and hepatocyte + endothelial cells) secreted albumin (h) and urea (i) for one week.

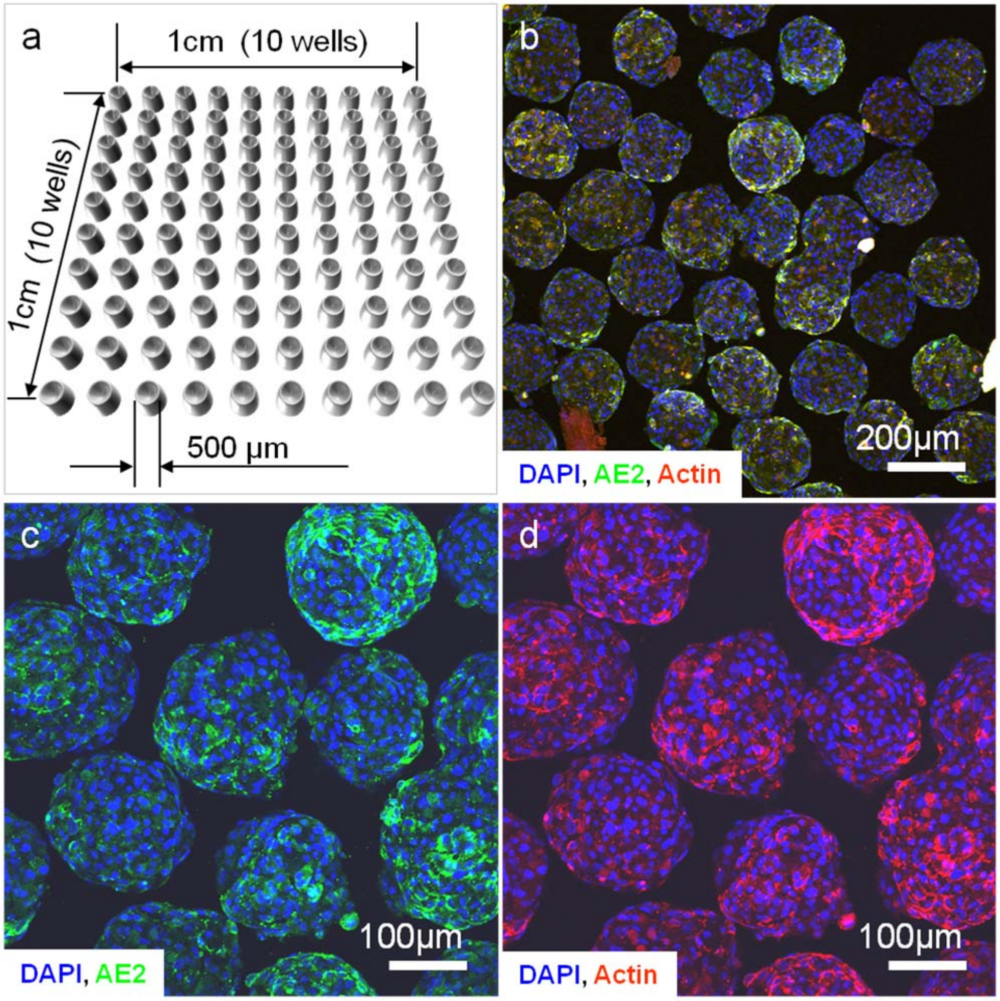

Supplementary Figure6. Spheroid culturing on the 10x10 concave microwell plate. (a) Schematic of the concave microwell arrayed plate for spheroidal formation. (b) Confocal microscope image of the hepatocyte-EC co-culturing in the concave microwell (blue, green and red indicate DAPI, Anion exchanger2 (AE2), and actin, respectively.). (c) Magnified confocal image of co-cultured spheroids stained with DAPI (blue) and AE2 (green). (d) Magnified confocal microscope image of the hepatocytes-EC spheroids stained with DAPI (blue) and actin (red).

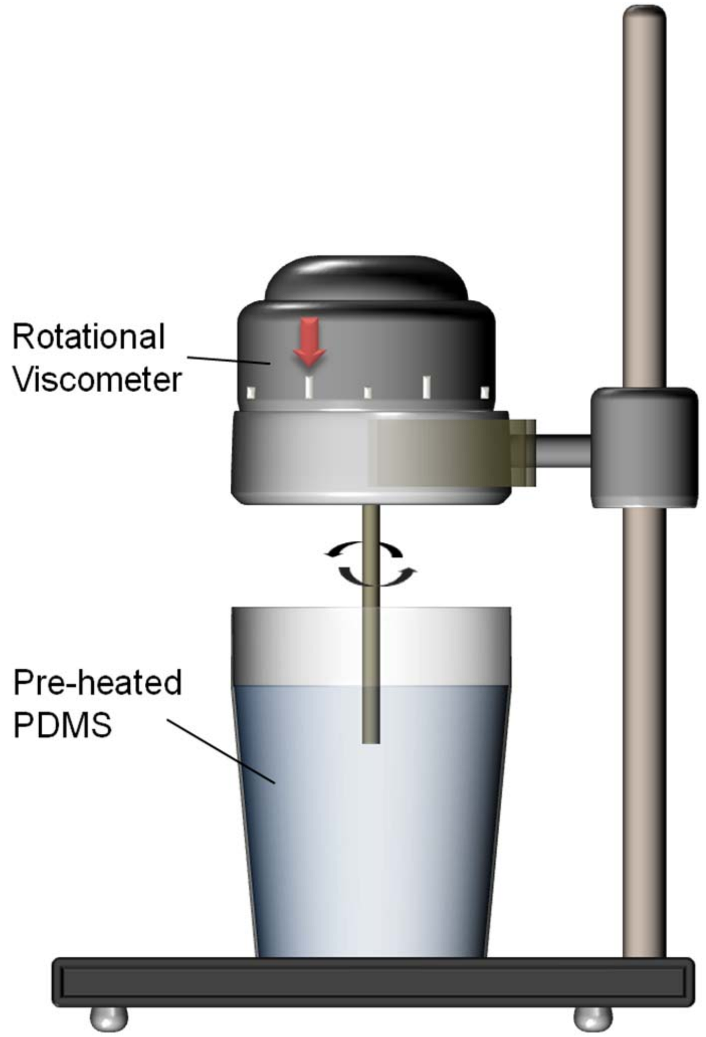

Supplementary Figure7. Measuring and preparing the viscosity of the HV-PDMS solution for fabricating the ultra-HAR structure.

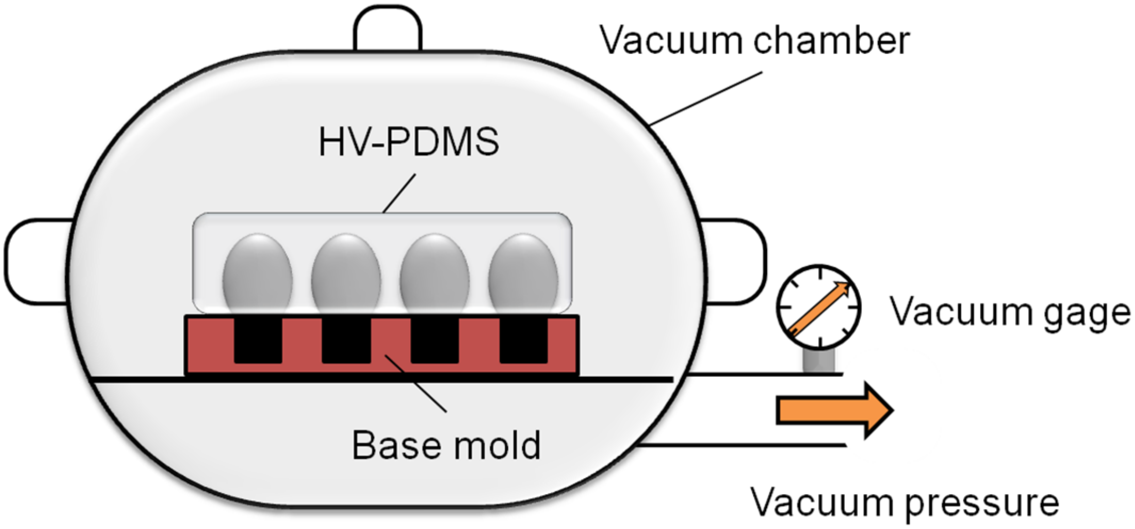

Supplementary Figure8. Schematic of the system used to fabricate the microstructures using viscoelastic lithography under a vacuum.

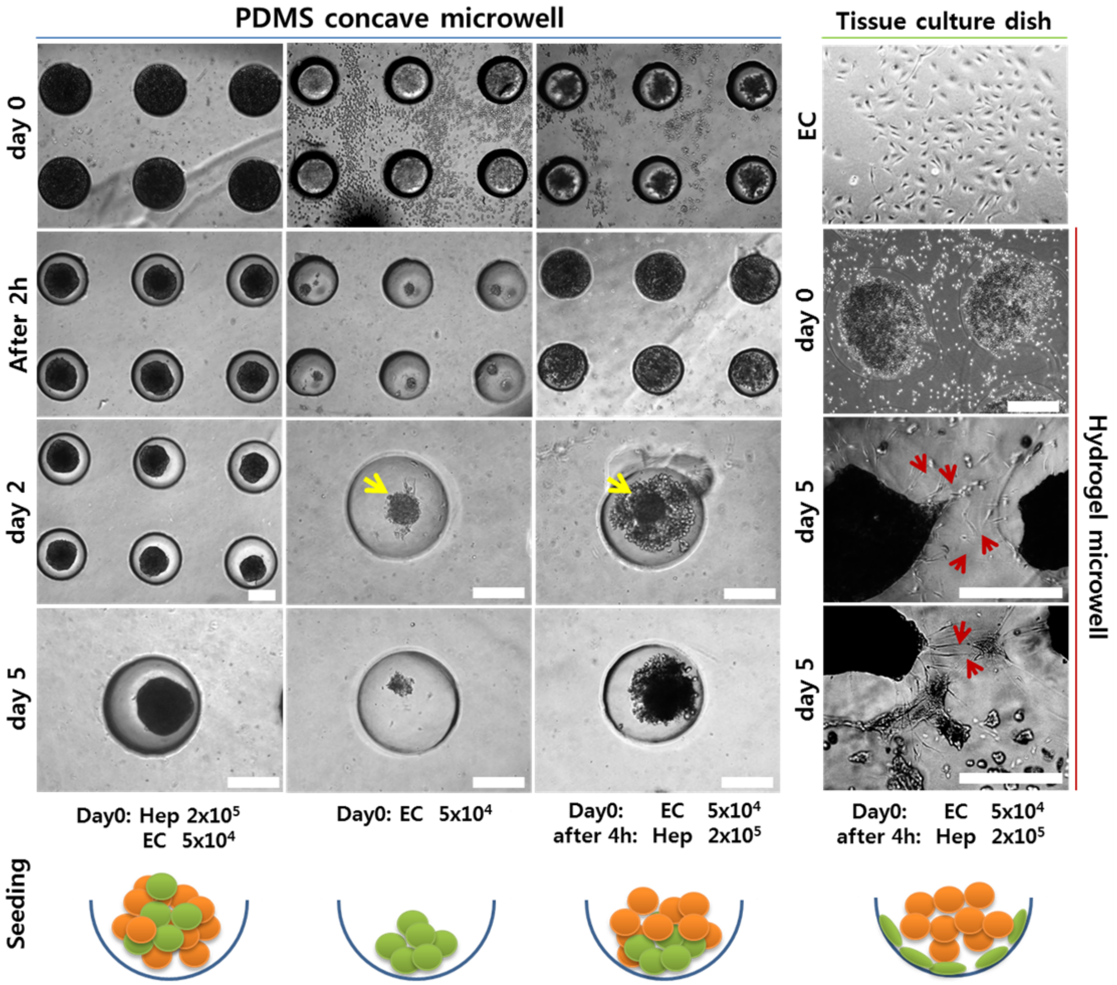

Supplementary Figure9. Microscopic images of co-culture spheroids in the PDMS microwell and in the hydrogel microwell. Yellow arrows indicate EC spheroid and red arrows indicate proliferated ECs forming vessel-like structures. Scale bars: 200μm

Supplementary Table 1. Primer design for reverse transcription PCR

Host: rat	Gene	Primer design	F/R	bp
Hepatic functions	Alb	ATCCTGAACCGTCTGTGTGT	F	121
		TCTCGTCAACTGTCAGAGCA	R	
	CYP2e1	GAGACCACCAGCACAACTCT	F	149
		TCCATGTAGGGCATATCCAG	R	
House gene	18s rRNA	CTGAGAAACGGCTACCACAT	F	115
		ATTACAGGGCCTCGAAAGAG	R	
**Host: human**	**Gene**	**Primer design**	**F/R**	**bp**
EC character	PECAM1	AAGGTAAACGGGAAGGAGATGT	F	118
		TCATGTTTGCCTAGCTCCCTA	R	
	vWF	TCTGGCTGAGGGAGGTAAAAT	F	117
		ACTTACAGCTTCCCACCTTGA	R	
Angiogenic function	MMP9	CCGGACCAAGGATACAGTTTG	F	114
		CAGTGAAGCGGTACATAGGG	R	
House gene	18s rRNA	GTAACCCGTTGAACCCCATT	F	151
		CCATCCAATCGGTAGTAGCG	R	

Supplementary Note 1

Calculation of the albumin and urea secretion

To counted cell number, we have used CyQUANT Cell Proliferation Assay Kit (Molecular Probes, Eugene, OR). For the experiment, hepatocyte spheroids were taken from the concave microwells, and dispersed to single cells using trypsin. For dispersion, hepatic spheroids were incubated with 0.25% trypsin at 37°C for 15 min. The cell pellet was frozen at -70°C, thawed at room temperature, and assayed with cell-lysis buffer and nuclear dye (200 μl/mold). The fluorescence of the samples was measured using a microplate reader (PerkinElmer, Akron, USA). The observed fluorescence was converted to cell number using a standard curve generated from hepatocytes. The number of cells per each method was 591±18 (n = 10). With this cell number, we have roughly calculated the secretion of albumin (2.68±0.11 μg/cell/day for co-culturing model and 2.53±0.15 μg/cell/day for mono-culturing model) and urea (77.5±1.32 ng/cell/day for co-culturing model and 66.28±2.18 ng/cell/day for mono-culturing model). We have calculated the normalized albumin and urea secretion of our previous study[1]. The albumin and urea secretion values for co-culturing model were calculated approximately respectively, and their values are 2.04μg/cell/day (albumin) and 42.18 ng/cell/day (urea).

Supplementary Reference

1. No, D. Y., Jeong, G. S. & Lee, S.-H. Immune-protected xenogeneic bioartificial livers with liver-specific microarchitecture and hydrogel-encapsulated cells. *Biomaterials* **35**, 8983-8991 (2014).
